# Matrix metalloproteases as maestros for the dual role of LPS- and IL-10-stimulated macrophages in cancer cell behaviour

**DOI:** 10.1186/s12885-015-1466-8

**Published:** 2015-06-05

**Authors:** Ana P. Cardoso, Marta L. Pinto, Ana T. Pinto, Marta T. Pinto, Cátia Monteiro, Marta I. Oliveira, Susana G. Santos, João B. Relvas, Raquel Seruca, Alberto Mantovani, Marc Mareel, Mário A. Barbosa, Maria J. Oliveira

**Affiliations:** 1 i3S-Instituto de Investigação e Inovação em Saúde/INEB-Institute of Biomedical Engineering, University of Porto, Porto, Portugal; 2 FEUP-Faculty of Engineering, University of Porto, Porto, Portugal; 3 ICBAS-Institute of Biomedical Sciences Abel Salazar, University of Porto, Porto, Portugal; 4 i3S-Instituto de Investigação e Inovação em Saúde/IPATIMUP-Institute of Molecular Pathology and Immunology of the University of Porto, Porto, Portugal; 5 i3S-Instituto de Investigação e Inovação em Saúde/IBMC-Institute for Cell and Molecular Biology, University of Porto, Porto, Portugal; 6 Department of Pathology and Oncology, Faculty of Medicine, University of Porto, Porto, Portugal; 7 Humanitas Clinical and Research Centre, Rozzano, Italy; 8 BIOMETRA Department, University of Milan, Milan, Italy; 9 Laboratory of Experimental Cancerology, Ghent University Hospital, Ghent, Belgium

**Keywords:** Tumour microenvironment, M1 and M2-like macrophages, Invasion, Angiogenesis, Gastrointestinal cancer, MMPs

## Abstract

**Background:**

The interactions established between macrophages and cancer cells are largely dependent on instructions from the tumour microenvironment. Macrophages may differentiate into populations with distinct inflammatory profiles, but knowledge on their role on cancer cell activities is still very scarce. In this work, we investigated the influence of pro-inflammatory (LPS-stimulated) and anti-inflammatory (IL-10-stimulated) macrophages on gastric and colorectal cancer cell invasion, motility/migration, angiogenesis and proteolysis, and the associated molecular mechanisms.

**Methods:**

Following exposure of gastric and colon cancer cell lines to LPS- and IL-10-stimulated human macrophages, either by indirect contact or conditioned media, we analyzed the effect of the different macrophage populations on cancer cell invasion, migration, motility and phosphorylation status of EGFR and several interacting partners. Cancer-cell induced angiogenesis upon the influence of conditioned media from both macrophage populations was assessed using the chick embryo chorioallantoic membrane assay. MMP activities were evaluated by gelatin zymograhy.

**Results:**

Our results show that IL-10-stimulated macrophages are more efficient in promoting *in vitro* cancer cell invasion and migration. In addition, soluble factors produced by these macrophages enhanced *in vivo* cancer cell-induced angiogenesis, as opposed to their LPS-stimulated counterparts. We further demonstrate that differences in the ability of these macrophage populations to stimulate invasion or angiogenesis cannot be explained by the EGFR-mediated signalling, since both LPS- and IL-10-stimulated macrophages similarly induce the phosphorylation of cancer cell EGFR, c-Src, Akt, ERK1/2, and p38. Interestingly, both populations exert distinct proteolytic activities, being the IL-10-stimulated macrophages the most efficient in inducing matrix metalloprotease (MMP)-2 and MMP-9 activities. Using a broad-spectrum MMP inhibitor, we demonstrated that proteolysis was essential for macrophage-mediated cancer cell invasion and angiogenesis.

**Conclusions:**

We propose that IL-10- and LPS-stimulated macrophages distinctly modulate gastric and colorectal cancer cell behaviour, as result of distinct proteolytic profiles that impact cell invasion and angiogenesis.

## Background

Solid tumours are complex entities with several cellular constituents other than malignant cells. Macrophages constitute a major component of the immune infiltrate in these tumours and are known to interact with cancer cells and to play a crucial role in distinct steps of cancer progression, such as survival, immune evasion, migration, invasion and metastasis [1–4]. The presence of macrophages is usually an indicator of poor prognosis in many types of malignancies [2, 5]. In colorectal cancer, tumour-associated macrophages (TAMs) have been frequently correlated with better prognosis [5, 6], although some studies refer that disease outcome may vary according to macrophage molecular profile and localization within the tumour [6–8]. In gastric cancer, few reports point TAMs as positive predictors of patient survival [9, 10], while most studies associate high macrophage densities with tumour promotion and worse overall survival [5, 11, 12]. Previous studies revealed that macrophages stimulate breast cancer cell migration and invasion through a paracrine loop involving colony-stimulating factor-1 (CSF-1), produced by cancer cells, and EGF produced by macrophages [1, 13]. Our own work recently reinforced these studies, describing that gastric and colorectal cancer cell motility, proteolysis and invasion are stimulated by macrophages and that epidermal growth factor (EGF) is a key molecule in this crosstalk [14]. However, nothing was described about the putative impact of different macrophage subpopulations in gastric and colorectal cancer cell properties and signalling.

Macrophages are highly plastic and very versatile in response to microenvironment stimuli, including cues released by neoplastic cells [15–17]. Despite their intermediate activation state, macrophages are generally classified into two main functional phenotypes, reflecting the Th1/Th2 response of CD4 T helper cells [18–20]. M1 macrophages are induced by interferon-gamma (IFN-γ), microbial products, such as lipopolysaccharides (LPS) and cytokines, like tumour necrosis factor-alpha (TNF-α) [21]. They are generally characterized by inflammatory, microbicidal and tumoricidal activities, high antigen presenting capacity, high secretion of IL-12, IL-23, IL-6, nitric oxide (NO) and reactive oxygen intermediates (ROI) and low IL-10 production [22]. On the other hand, M2 and M2-like macrophages polarize in response to IL-4 and IL-13, IL-10 or glucocorticoid hormones and are generally described to present low IL-12 and IL-6 and high IL-10 expression, as well as an increased ability to scavenge, repair and remodel tissue, promoting angiogenesis and tumour progression [22, 23]. Although most current studies suggest TAMs as being a skewed M2-like macrophage population, engaging in cancer promoting activities, their phenotype can vary according to their distribution within the tumour [19, 24–26]. Therefore, clarifying the role of distinct macrophage subsets in cancer and unravelling the concomitant molecular mechanisms will contribute to the identification of novel therapeutic targets and biomarkers useful for patient stratification.

In the present work, we studied LPS- and IL-10-stimulated macrophages modulation of gastric and colorectal cancer cell-related activities, such as invasion, proteolysis, motility, migration and angiogenesis and determined the associated molecular mechanisms. Overall, our results demonstrate that distinct proteolytic activities of these macrophage populations differently modulate the behaviour of gastric and colorectal cancer cells, providing new insights for the development of new and more efficient anti-tumour therapies.

## Methods

### Cell culture and reagents

AGS (CRL-1739) and RKO (CRL-2577) cells, derived respectively from a human diffuse gastric and colon carcinoma, were purchased from the American Type Culture Collection (ATCC, Manassas, VA, USA) in 2012. Cell lines were tested and authenticated by autosomal STR DNA profiling, in which a DNA sample was analysed with POWERPLEX 16 HS kit (Promega, Madison, WI, USA). The cell lines were last tested and authenticated on May 20^th^ 2014, by a laboratory accredited by the College of American Pathologists and with a Quality Management System certified in accordance with NP EN ISO 9001:2008 (IPATIMUP Diagnostics, Porto, Portugal). Cells were cultured at 37 °C and 5 % CO_2_ humidified-atmosphere in RPMI1640 medium (Invitrogen, Merelbeke, Belgium), supplemented with 10 % fetal bovine serum (FBS) (Lonza, Basel, Switzerland), 100 U/ml penicillin and 100 μg/ml streptomycin (Invitrogen).

### Human monocyte isolation and macrophage differentiation

Human monocytes were isolated from healthy blood donors as previously described [14]. For monocyte to macrophage differentiation, 10^6^ monocytes/ml/3,8cm^2^ were then cultured for 10 days in RPMI1640 medium, supplemented with 10 % FBS and 100 U/ml penicillin and 100 μg/ml streptomycin, in absence of M-CSF or other exogenous factors. LPS- and IL-10-stimulated macrophages were obtained by adding 10 ng/ml LPS (Sigma-Aldrich) or IL-10 (ImmunoTools, Friesoythe, Germany), respectively, for additional 72 h. Unstimulated (naïve) macrophages were maintained with renewed medium and used as control. All experimental protocols were conducted following the approval and recommendations of the Ethics Comittee for Health from Centro Hospitalar S. João (Porto – References 259/11 and 260/11).

### Flow cytometry

For cell surface receptor expression analysis, unstimulated, LPS- and IL-10-stimulated macrophages were harvested, by incubation with PBS-5 mM EDTA, for 30 min at 37 °C. Macrophages were then resuspended in FACS buffer (PBS, 2 % FBS, 0.01 % sodium azide), and stained with anti-human CD14-FITC, HLA-DR-PE (ImmunoTools) and CD163-PE (R&D Systems, Minneapolis, MN, USA), for 30 min at 4 °C in the dark. Isotype-matched antibodies were used as negative controls, to define background staining. Cells were acquired on a FACSCalibur™ Flow Cytometer (BD Biosciences), using Cell Quest Software (collecting 10 000 cells). Analysis was performed with FlowJo software. Percentage of positive cells was calculated by subtracting the respective isotype control. Experiments were performed with cells from at least five different donors.

### Enzyme**-**linked immunosorbent assay (ELISA)

TNF-α, IL-6 and IL-10 cytokines, present in conditioned media (CM) from unstimulated, LPS- and IL-10-stimulated macrophages, were quantified by ELISA according to manufacturer’s instructions (BioLegend, San Diego, CA, USA) [14].

### Invasion assays

Invasion assays were performed as previously [14], using BD BioCoat*™* Matrigel*™* Invasion Chambers (BD Biosciences, Madrid, Spain) and AGS or RKO cells in the upper compartment, and LPS- (LPSmac) or IL-10-stimulated macrophages (IL-10mac) in the lower compartment. To discard any influence of soluble factors released along macrophage differentiation, media was renewed before invasion assays. The broad MMP inhibitor Galardin (Calbiochem, Nottingham, UK) was used at a final concentration of 10 μM. The invasive ratio was calculated as the ratio between the number of invasive cells in the test condition and the number of invasive cells in the control condition.

### Conditioned media preparation

At the end of Matrigel™ invasion assays, CM of cancer cells (CMMat(AGS)), LPS- (CMMat(LPSmac)) or IL-10-stimulated (CMMat(IL-10mac)) macrophages or cancer cells cultured in the presence of LPS- (CMMat(AGS + LPSmac)) or IL-10-stimulated (CMMat(AGS + IL-10mac)) macrophages were collected. The influence of soluble factors produced by LPS- (CM(LPSmac)) and IL-10-stimulated (CM(IL-10mac)) macrophages, in the absence of ECM components (without Matrigel™) were also prepared.

### Immunocytochemistry

To evaluate macrophage morphology and cytoskeleton organization, 19×10^4^ monocytes/cm^2^ were seeded on glass coverslips upon isolation, and left for 10 days in culture. Treatments with LPS and IL-10 were performed as described above. To investigate the effect of distinct macrophage populations on cancer cell motility and EGFR phosphorylation, 2.7×10^4^ AGS cells/cm^2^, seeded on glass coverslips and maintained at 37 °C, 5 % CO_2_, were treated or not with CM from LPS- (CM(LPSmac)) or IL-10-stimulated macrophages (CM(IL-10mac)) for 1 or 6 h. In parallel, RPMI media (RPMI) was used as control. Cells were immunostained for phosphoEGFR (Tyr1086), α-tubulin and F-actin and analysed as previously described [14].

### Calculation of macrophage aspect ratio

Macrophage aspect ratio was quantified using ImageJ software on images of actin/tubulin unstimulated, LPS- or IL-10-stimulated macrophages. Aspect ratio was calculated as the quotient between the length of each cell major and minor axes, as previously described [27]. At least 100 cells per donor/per condition were scored, and at least three independent experiments were analysed with cells from three different donors.

### Quantification of motility-associated structures

Filopodia, lamellipodia and stress fibers were quantified using ImageJ software on images of actin/tubulin regarding AGS cells treated for 6 h with RPMI or CM from LPS- or IL-10-stimulated macrophages. The percentage of cells with these structures was calculated considering the total cell number. At least 100 cells per donor/per condition were scored, and at least three independent experiments were analysed with cells from three different donors

### Timelapse microscopy

To determine the effect of LPS- and IL-10-stimulated macrophages on cancer cell migration, 5×10^4^ AGS cells/cm^2^ were seeded. Immediately before each experiment, cells were treated with CM from LPS- (CM(LPSmac)) or IL-10-stimulated (CM(IL-10mac)) macrophages (1/3 total volume) or equivalent RPMI medium (RPMI), as control. Cell trajectories followed for 13 h were quantified as previously described [14].

### Gelatin zymography

MMP activity of LPS- and IL-10-stimulated macrophages, and of co-cultures of AGS cells with both macrophage populations was investigated by analysing CM from invasion assays through gelatin zymography, as previously described [14, 28].

### Angiogenesis assay

Fertilized chicken (*Gallus gallus*) eggs obtained from commercial sources (Pintobar, Braga, Portugal) were incubated at 38 °C. At day 3 of incubation, a window was opened in the shell, and 2–2.5 ml albumen was removed. The window was sealed with adhesive tape, and the egg re-incubated. At day 10 of incubation, a 3 mm silicon ring was placed on the growing chorioallantoic membrane (CAM), under sterile conditions. Then, 1×10^6^ AGS cells in RPMI medium and 1×10^6^ AGS cells in CM from LPS-treated (CM(LPSmac)) or IL-10-stimulated macrophages (CM(IL-10mac)), with or without Galardin (30 μM), were incubated within two separate rings. The window was resealed, and 72 h after inoculation, rings were removed, and the CAM was excised and photographed *ex ovo* under a stereoscope (Olympus; SZX16 coupled DP71 camera). The number of new vessels (<20 μm diameter) growing radially towards the ring area was counted. At least 16 eggs were used for each condition. Means of ratios between vessel number in the test condition and vessel number in the control condition of each animal ± standard error mean (SEM) were evaluated, and the statistical significance of the differences was determined using the Student’s *t* test (for samples with unequal variance).

### Western blot

AGS or RKO cells, at approximately 80 % confluency, were serum-starved overnight and treated with CM from unstimulated (CM(mac)), LPS- (CM(LPS-mac)), IL-10-stimulated macrophages (CM(IL-10mac)) or RPMI as control (CMRPMI) for 1 h. Cell lysates, electrophoresis and immunoblotting were performed as previously described [14]. Primary antibodies used included rabbit polyclonal antibodies against phospho-EGFR(Y^1086^) (Zymed-Invitrogen), phospho-Src(Y^416^), phospho-ERK1/2(T^202^/Y^204^), phospho-p38(Thr^180^/Tyr^182^), phospho-Akt(S^473^), Akt, ERK1/2, Src, p38 (Cell Signaling, MA, USA), α-tubulin (Sigma-Aldrich) or mouse monoclonal antibody against EGFR (Transduction). Donkey anti-rabbit or sheep anti-mouse*-*HRP-conjugated secondary antibodies (GE Healthcare) were used, followed by ECL-Detection (GE Healthcare).

### siRNA transfection

siRNA targeting EGFR, previously validated for knockdown efficiency in AGS, was purchased from Invitrogen. Prior to transfection, AGS cells at 60 % confluence were incubated in serum–antibiotic-free RPMI1640. Cells were transiently transfected (75 nM EGFR siRNA) using Lipofectamine2000 transfection reagent (Invitrogen). As negative control, cells transfected with Lipofectamine2000 were used. Eight hours after transfection, medium was replaced by RPMI1640 supplemented with 10 % FBS. Knockdown efficiency was tested by western blot, 48 h after transfection.

### Statistical analysis

Data were analysed using GraphPad Prism v.5 software, and expressed as mean values of at least three independent experiments and ± Standard Deviations (SD) or Standard Error Mean (SEM), as indicated. Differences were tested with Mann-Whitney test or Student’s *t* test for non-parametric data, and were considered significant at a *p* value of less than 0.05. Experiments were performed with at least three different blood donors, as indicated.

## Results

### LPS- and IL-10-stimulated macrophages present distinct phenotypes

**Fig. 1:**
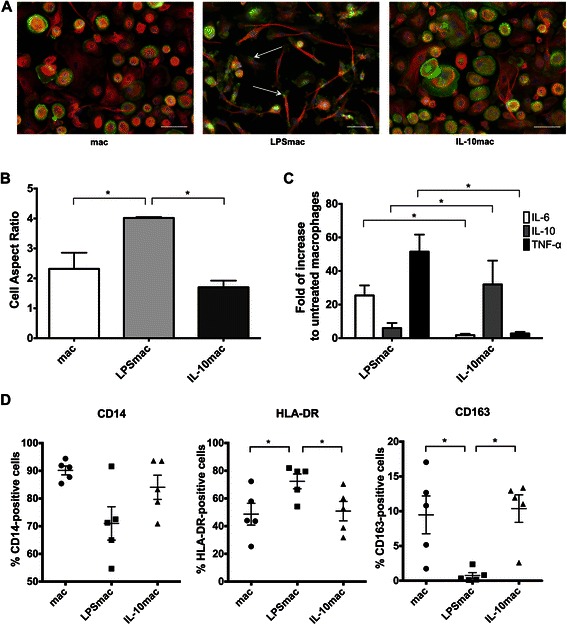
Phenotypic characterization of LPS- and IL-10-stimulated macrophages derived from human CD14^+^ peripheral blood monocytes. **a** Representative images of actin and tubulin stainings of LPS- and IL-10-stimulated macrophages polarized in absence of other external stimuli (mac) or in the presence of 10ng/ml LPS (LPSmac) or IL-10 (IL-10mac), respectively. F-actin was stained with Phalloidin-FITC (green), α–tubulin with a specific monoclonal antibody followed by incubation with AlexaFluor594 secondary antibody (red) and nuclei were counterstained with DAPI (blue). Scale bars represent 50 μm. **b** Morphological differences between macrophage populations were quantified by calculating the cell aspect ratio (quotient between cell major and minor axes) of actin/tubulin stained cells. Chart reflects measurements of at least 100 cells per donor from, at least, 3 distinct donors. Bars represent mean values and flags indicate standard deviations. **c** Cytokine production profile of LPS- and IL-10-stimulated macrophages. Cytokine concentration was measured by ELISA in conditioned media from distinct macrophage populations. Charts indicate fold increase in IL-6, IL-10 and TNF-α expression, in comparison to unstimulated macrophages. Data is representative of the cytokine profile of cells derived from at least 7 different donors. Bars represent mean values and flags indicate standard deviations. **d** Expression of typical macrophage lineage (CD14) and polarization markers (HLA-DR and CD163) was determined by flow cytometry of unstimulated, LPS- and IL-10-stimulated macrophages. Scatter charts represent percentage of positive cells for each cell surface marker considering data obtained with cells derived from 5 different donors. *, significantly different at *p* < 0.05. IL-10, interleukin-10; LPS, lipopolysaccharide

To study the role of distinct macrophage populations on the modulation of cancer cell-related activities, primary human monocytes were first differentiated into macrophages, and left unstimulated or stimulated with 10 ng/ml of LPS (LPS-stimulated) or IL-10 (IL-10-stimulated), respectively. Macrophage polarization into distinct populations was confirmed by morphology, actin-tubulin cytoskeleton organization, cell surface receptors and cytokine secretion analysis. F-actin and α-tubulin staining evidenced differences in cytoskeleton organization between LPS- and IL-10-stimulated macrophages (Fig. 1a). LPS-stimulated macrophages were elongated, presenting a significantly higher cell aspect ratio (quotient between cell major and minor axes length) (4.02 ± 0.03) (Fig. 1b), in comparison with IL-10-stimulated (1.70 ± 0.23) or with unstimulated macrophages (2.32 ± 0.54). Unstimulated macrophages constituted a more morphologically heterogeneous population than those stimulated with LPS, which contained areas of pronounced actin staining along the cell body (Fig. 1a, arrows), or those stimulated with IL-10, which contained podosome-like actin protrusions displayed along the entire cell periphery (Fig 1a and b).

The macrophage cytokine profile, performed by ELISA, revealed that LPS-stimulated macrophages produced significantly higher levels of IL-6 and TNF-α, in comparison with IL-10-stimulated ones, which in turn, secreted significantly higher IL-10 and lower IL-6 and TNF-α levels (Fig. 1c). Flow cytometry analysis of macrophage surface receptors revealed that CD14, a macrophage lineage marker, was not statistically differently expressed between all macrophage populations (Fig. 1d, left panel). As expected, LPS-stimulated macrophages presented significantly higher expression of the M1-like marker HLA-DR and lower of the M2-like marker CD163 (Fig. 1d, middle panel), in contrast to IL-10-stimulated macrophages, which exhibited significantly lower expression of HLA-DR and higher of CD163 (Fig. 1d, right panel).

Our results are in accordance with those previously described in the literature concerning cytokine and cell surface receptor expression profiles of M1- and M2-like macrophages [29–31].

### IL-10-stimulated macrophages are more efficient in stimulating gastric and colorectal cancer cell invasion

**Fig. 2:**
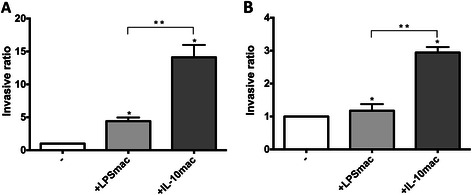
IL-10-stimulated macrophages are more efficient in stimulating cancer cell invasion. **a** AGS human gastric or **b** RKO human colorectal cancer cells were incubated in BD BioCoat^TM^ Matrigel^TM^ Invasion Chambers for 24 h with RPMI medium (-), or human macrophages differentiated for 10 days and stimulated for 72 h with 10 ng/ml LPS (LPSmac) or 10 ng/ml IL-10 (IL-10mac). Invasive cells and invasive ratio were determined as described in Materials and Methods. Bars represent mean values of independent experiments performed with, at least, 4 different donors; flags indicate standard deviations

To evaluate the influence of distinct macrophage populations on gastric and colorectal cancer cell invasion, we performed Matrigel invasion assays confronting non-invasive gastric (AGS) or colorectal cancer (RKO) cells with human macrophages, polarized towards an M1 or an M2-like phenotype (Fig. 2a and b). Interestingly, the presence of IL-10-stimulated macrophages significantly increased AGS invasion through Matrigel-coated filters, relatively to AGS cells alone and to LPS-stimulated macrophages. The latter were still effective in inducing AGS cell invasion (fold of increase 4.41 ± 0.56), although to a significantly lower extent than IL-10-stimulated macrophages (14.11 ± 1.89) (Fig. 2a). The observed stimulation of invasion seems to occur by the action of one or more soluble factors since, in this experimental system, the two cell types do not contact directly. Similarly, IL-10-stimulated macrophages induced RKO invasion (fold of increase 2.935 ± 0.20) in a higher extent than LPS-stimulated macrophages (1.203 ± 0.24) (Fig. 2b). As controls, 10 ng/ml LPS or 10 ng/ml IL-10 alone were added to invasion assays in the absence of macrophages, having no influence on the number of invasive cancer cells (data not shown).

Altogether, these results indicate that IL-10- and LPS-stimulated macrophages affect gastric and colorectal cancer cell invasion in different extents, being the IL-10-stimulated more efficient. Since AGS cells were more susceptible to macrophage-mediated invasion than RKO, as indicated by the invasive ratios obtained, these cells were selected for subsequent studies.

### IL-10-stimulated macrophages are more efficient in inducing cancer cell motility and migration

**Fig. 3:**
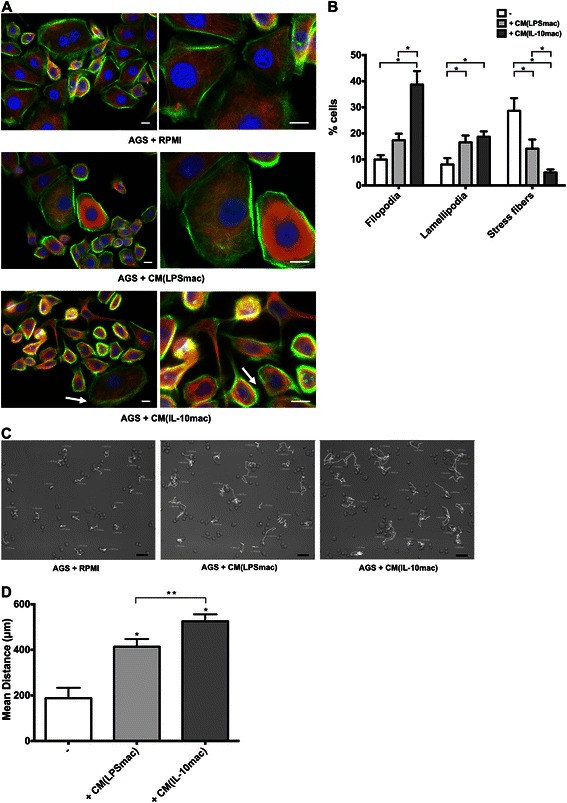
IL-10-stimulated macrophages are more efficient in stimulating cancer cell motility and migration. **a** Representative images of actin and tubulin stainings of AGS cells incubated, during 6 h, with RPMI (AGS + RPMI), LPS- (AGS + CM(LPSmac)) or IL-10-stimulated macrophage conditioned medium (AGS + CM(IL-10mac)). F-actin was stained with Phalloidin-FITC (green), α-tubulin with a specific monoclonal antibody following incubation with an AlexaFluor594 secondary antibody (red) while nuclei were counterstained with DAPI (blue). Scale bar represents 10 μm. **b** Quantification of motility-associated actin/tubulin structures on AGS cells treated for 6 h with RPMI (-) or CM from LPS- (+CM(LPSmac)) and IL-10- (+CM(IL-10mac)) stimulated macrophages. % of cells with filopodia, lamellipodia or stress fibers was calculated relatively to total cell number on images of F-actin/α-tubulin staining. Bars represent mean values obtained with at least 100 cells in independent experiments with CM of macrophages obained from at least 3 different blood donors; flags indicate standard error mean. *, significantly different at *p* < 0.05. **c** Representative images of AGS cell trajectories followed for 13 h, using timelapse microscopy. Cells were incubated in the presence of RPMI (AGS + RPMI) or CM from LPS- (AGS + CM(LPSmac)) or IL-10-stimulated macrophages (AGS + CM(IL-10mac)). Trajectories are represented as white lines traced between initial, intermediate and final *xy* positions. Scale bar represents 50 μm. **d** Distance (μm) travelled by AGS cells in the presence of RPMI (AGS) or CM from LPS- (CM(LPSmac)) or IL-10-stimulated macrophages (CM(IL-10mac)) was quantified using the LSMib Zeiss software (Carl Zeiss, Aalen, Germany) and bars represent mean values of distance migrated. A minimum of 100 cell trajectories were measured in independent experiments, with CM from macrophages of at least 5 different donors; flags indicate standard deviations. *, significantly different from AGS or RKO in RPMI medium at *p* < 0.05; **, significantly different at *p* < 0.05

To investigate the effect of IL-10- and LPS-stimulated macrophages on cancer cell motility and cytoskeleton organization, F-actin and α-tubulin stainings were conducted on AGS cells, after 6 h-treatments with CM from both macrophage populations or with control RPMI medium. In response to soluble factors produced by both IL-10- and LPS-stimulated macrophages, motility-associated structures, such as filopodia and lamellipodia, were observed (Fig. 3a, arrows). These actin-rich structures, essentially filopodia, were more frequent on cells exposed to CM from IL-10-stimulated (CM(IL-10mac)) (38.7 ± 5.2 %), than on cells exposed to CM from LPS-stimulated macrophages (CM(LPSmac)) (17.3 ± 2.5 %) or to control RPMI (9.9 ± 1.7 %) (Fig. 3b). In addition, cells exposed to CM from IL-10-stimulated macrophages had a more elongated shape, than cells exposed to RPMI only (AGS + RPMI). The latter presented a pronounced polyhedral shape and a strong cortical actin staining at the periphery (Fig. 3a). Aiming to study the effect of both macrophage populations on cancer cell migration, high-resolution timelapse microscopy was performed for 13 h. In the presence of CM from IL-10-stimulated macrophages, cancer cells described wider trajectories (Fig. 3c) and travelled significantly longer distances (Fig. 3d) in comparison to cancer cells stimulated with CM from LPS-stimulated macrophages (CM(LPSmac)). Overall these results indicate that IL-10-stimulated macrophages are more efficient than LPS-sitmulated macrophages in inducing cancer cell migration.

### IL-10-stimulated macrophages promote cancer cell mediated-angiogenesis

**Fig. 4:**
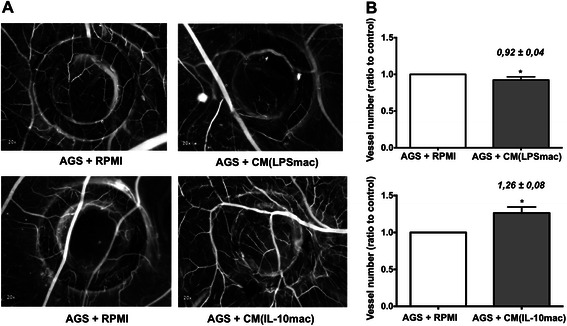
IL-10-stimulated macrophages promote cancer cell angiogenic response in the chick embryo chorioallantoic membrane (CAM) assay. **a** Representative images of the CAM showing AGS cell inoculation area (ring delimited). A control with AGS and RPMI (AGS + RPMI) was always included in each egg (20 X magnification), next to the inoculation site of AGS with CM from LPS- (AGS + CM(LPSmac)) or IL-10-stimulated (AGS + CM(IL-10mac)) macrophages. **b** Quantification of the number of new blood vessels grown towards each inoculation area (only vessels <20 μm diameter were counted). This quantification is compared with the control condition (AGS + RPMI) present at each egg (ratio between the vessel number in the test condition and the vessel number in the control condition). Bars represent mean values obtained with 18 eggs for AGS + CM(LPSmac), and 16 eggs for AGS + CM(IL-10mac) and flags indicate standard error mean *, significantly different at *p* <0.05

Angiogenesis is known to be crucial in several steps of tumour progression and M2-like macrophages are described to have pro-angiogenic potential [32]. To explore the influence on new vessels formation of IL-10- and LPS-stimulated macrophages, we incubated AGS cells with CM from both populations (AGS + CM(LPSmac) and AGS + CM(IL-10mac), respectively) in the chick embryo chorioallantoic membrane (Fig. 4a). As internal control, AGS cells with RPMI medium (AGS + RPMI) were included in each egg. Our results show that inoculation of AGS cells with CM from IL-10-stimulated macrophages resulted in a significantly higher number of new vessels, growing outwards the inoculation area (Fig. 4b, bottom chart). This indicates that soluble factors produced by this specific macrophage population are stimulating cancer cell-mediated angiogenesis. Conversely, CM from LPS-stimulated macrophages decreased the angiogenic response, since the number of new vessels formed was lower than in the control (Fig. 4b, upper chart). Altogether, our results suggest that molecules produced by IL-10-stimulated macrophages switch the balance of pro- and anti-angiogenic molecules towards the stimulation of angiogenesis, while unstimulated and LPS-stimulated macrophages have the opposite effect.

### Cancer cell invasion requires activation of EGFR signalling in the presence of IL-10- and LPS-stimulated macrophages

**Fig. 5:**
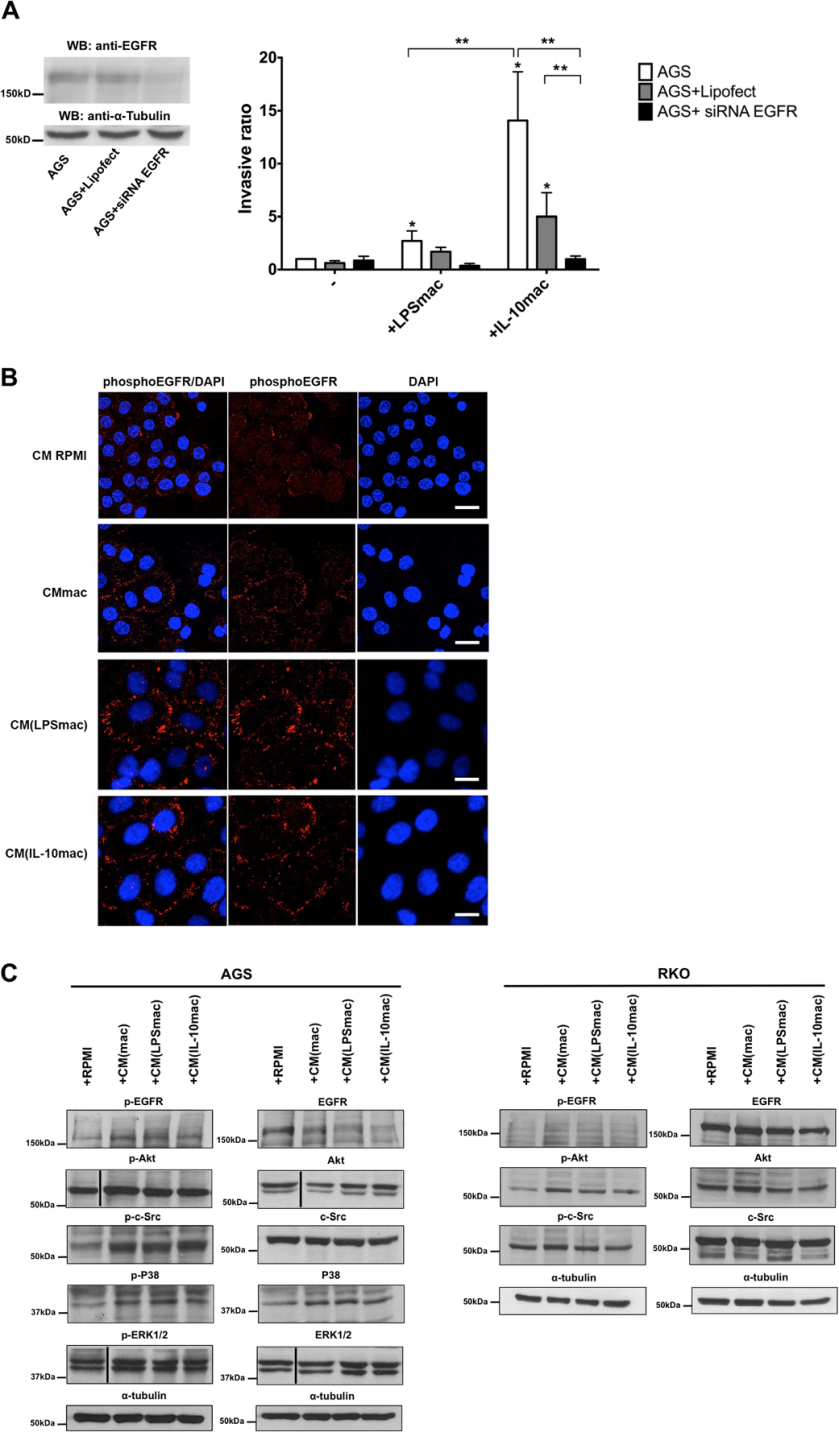
EGFR is required for both LPS- and IL-10-stimulated macrophage-mediated gastric cancer cell invasion. **a** EGFR expression was transiently silenced by transfection with validated siRNA, at 48 h post-transfection. **b** Invasion assays with AGS cells (AGS), AGS cells with Lipofetamine2000 (AGS + Lipofect) or with Lipofectamine2000 transfected together with siRNA directed to EGFR (AGS + siRNA EGFR) were performed in the presence or absence of LPS- (LPSmac) or IL-10-stimulated (IL-10mac) macrophages. These assays were conducted at 24 h post-transfection and stopped at 48 h post-transfection, when maximum inhibition was achieved. Bars represent mean values of independent experiments performed with macrophages from at least 4 different donors and flags indicate standard error mean * significantly different from AGS at *p* < 0.05; ** significantly different at *p* < 0.05. **c** Tyrosine phosphorylation status of EGFR residue Y^1086^ (red) after 1 h of incubation of AGS cells with RPMI (CM RPMI) or CM from unstimulated (CMmac), LPS- (CM(LPSmac)) or IL-10-stimulated (CM(IL-10mac)) macrophages. Nuclei were counterstained with DAPI (blue). Scale bar represents 10 μm. The image is representative of independent experiments performed with CM of macrophages, from at least three different donors. **d** AGS or RKO cells were treated or not (RPMI), during 1 h, with CM from unstimulated (CMmac), LPS- (CM(LPSmac)) or IL-10-stimulated (CM(IL-10)mac) macrophages. Cell lysates were immunoblotted for phosphorylated and total EGFR (Y^1086^), c-Src (Y^416^), Akt (S^473^), ERK1/2 (T^202^/Y^204^), and p38 (Thr^180^/Tyr^182^). Immunoblots were analyzed by densitometry analysis in comparison with corresponding α-tubulin and total protein expression levels. Images are representative of independent experiments performed with CM of macrophages from at least 3 different donors

EGFR and its associated signalling molecules have been previously implicated in macrophage-derived stimuli of invasion. In fact, silencing cancer cell EGFR expression or immunodepletion of EGF from macrophage CM, led to inhibition of motility, and abrogation of cancer cell invasion [14]. Therefore, to evaluate if EGFR is also necessary for IL-10- and LPS-stimulated macrophage-mediated invasion, AGS cells transiently silenced with siRNA for this receptor were confronted with both macrophage populations on Matrigel invasion assays. Since silencing of EGFR reached its maximum 48 h post-transfection (Fig. 5a), invasion assays were performed 24 h after transfection. A significant decrease in the invasion ability of cancer cells confronted with IL-10-stimulated macrophages was observed upon EGFR silencing, even when comparing with AGS cells transfected with Lipofectamine only. The same inhibitory effect was observed for LPS-stimulated macrophages, although not significantly, probably due to the lower levels of invasion already induced by this macrophage population (Fig. 5a).

Taking into account that EGFR is required for IL-10- and LPS-stimulated macrophage-mediated invasion, we evaluated if both macrophage populations differently affected cancer cell EGFR signalling. Interestingly, no differences in terms of EGFR tyrosine phosphorylation were found among AGS cells treated with CM from these distinct macrophage populations (Fig. 5b). Comparable patterns of intense membrane phosphoEGFR immunostaining were observed, whereas in the control condition (AGS + RPMI), the levels of phosphorylation were low and scattered throughout the cytoplasm. To evaluate the effect of distinct macrophage populations on the activation of EGFR signalling partners, lysates of AGS or RKO cells, previously incubated or not with CM from unstimulated (CMmac), LPS- (CM(LPSmac)) and IL-10-stimulated macrophages (CM(IL-10mac)), were analysed by immunoblotting. Our results indicate that, after 1 h, the three macrophage populations induce similar increases in the phosphorylation of EGFR (Υ^1086^), c-Src (Υ^416^), ERK1/2 (T^202^/Υ^204^), AKT (S^473^) and p38 (T^180^/T^182^) (Fig. 5c, left panel) in AGS cells. Since no differences in activation were observed in gastric cancer cells, similar studies were conducted with colorectal cancer cells. Similar results were obtained in terms of RKO cells phosphorylation of EGFR (Υ^1086^), c-Src (Υ^416^) and AKT (S^473^), upon treatments with CM from unstimulated, LPS- and IL-10-stimulated macrophages (Fig. 5c, right panel). Altogether, these results point EGFR signalling as fundamental for the induction of invasion provided by IL-10- and by LPS-stimulated-macrophages, as silencing EGFR in AGS cells caused them to be unresponsive to stimulation of invasion from both macrophage subsets. This effect is more prominent in terms of IL-10-treated macrophages stimulation because LPS-treated macrophages already cause lower levels of AGS invasion even with intact EGFR expression. These observations led us to the conclusion that other factors might be responsible for the distinct pro-invasive ability of both macrophage populations.

### Macrophage-mediated invasion and angiogenesis are dependent on MMP activity

**Fig. 6:**
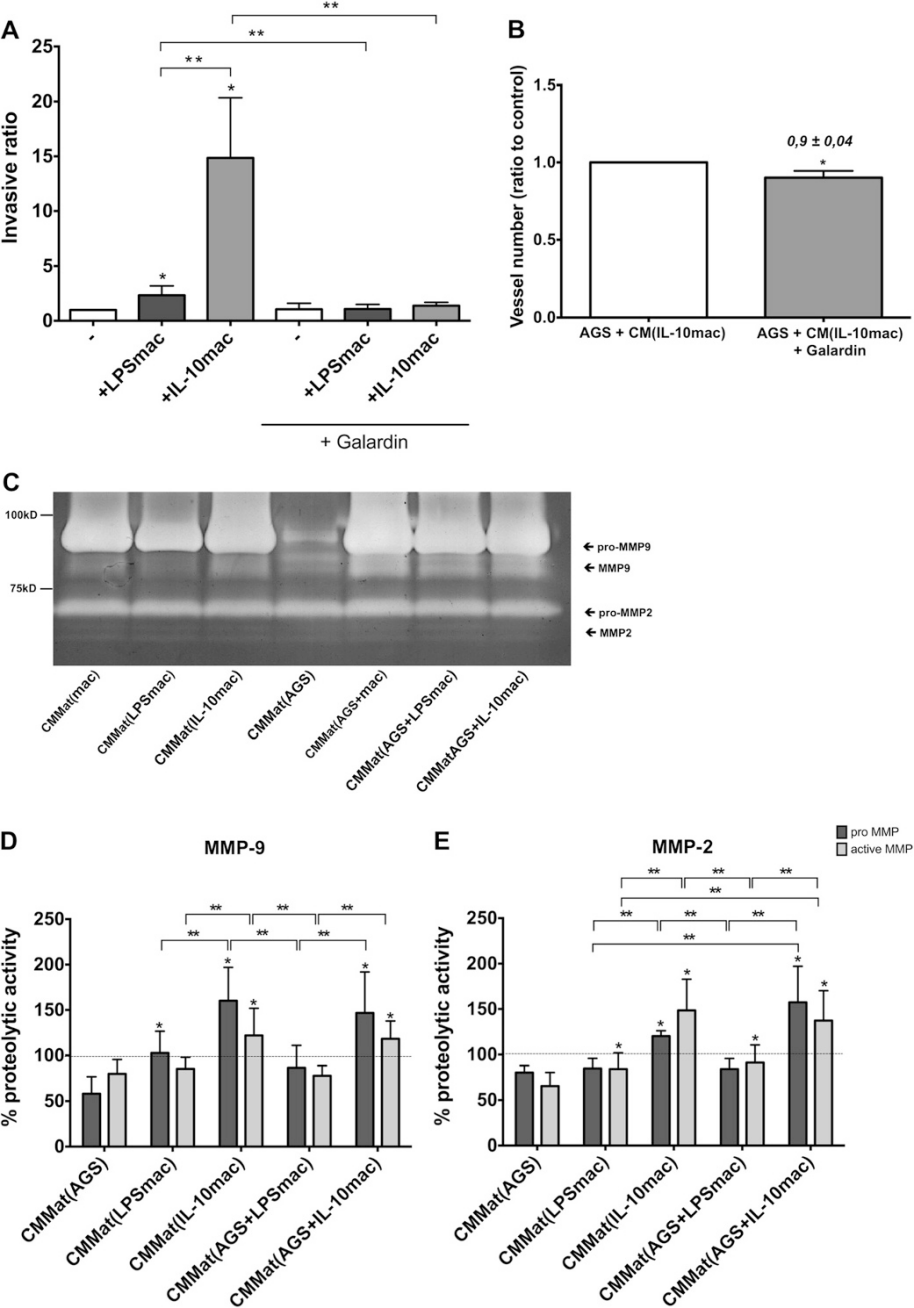
MMP activity influences LPS- and IL-10-stimulated macrophage-mediated cancer cell invasion and angiogenesis. **a** AGS cells were incubated in BD BioCoat^TM^ Matrigel^TM^ Invasion Chambers for 24 h with RPMI medium (-) or macrophages stimulated for 72 h with 10 ng/ml LPS (LPSmac)) or with 10 ng/ml IL-10 (IL-10mac) and supplied or not with a pharmacological inhibitor of matrix metalloproteases, Galardin (10 μM). Invasive cells were determined as described in Materials and Methods. Bars represent mean values of independent experiments performed with, at least, 4 different donors; flags indicate standard deviations. *, significantly different from AGS in RPMI medium at *p* < 0.05; **, significantly different at *p* < 0.05. **b** Quantification of the number of new blood vessels grown in the CAM towards each inoculation area (<20 μm diameter). A control with AGS and CM from IL-10-treated macrophages without Galardin (AGS + CM(IL-10mac)) was always included in each egg next to the inoculation area of AGS and CM of IL-10-stimulated macrophages supplemented with Galardin (30 μM) (AGS + CM(IL-10mac) + Galardin). Bars represent mean values (ratio between the vessel number in the test condition and the vessel number in the control condition, per animal) obtained from a total of 35 eggs and CM of macrophages derived from 3 different donors. Flags indicate standard error mean; *, significantly different at *p* <0.05. **c** Conditioned media from Matrigel^TM^ invasion assays containing AGS (CMMat(AGS)), unstimulated (CMMat(mac)), LPS- (CMMat(LPSmac)), IL-10-stimulated macrophages (CMMat(IL-10mac)), AGS and unstimulated (CMMat(mac)), LPS- (CMMat(AGS + LPSmac)) and IL-10-stimulated macrophages (CMMat(AGS + IL-10mac)) were run on gelatin zymograms. Proteolytic activity bands were revealed in white on a blue background stained with Coomassie. **d** and **e** Densitometry analysis using QuantityOne® software (BioRad) allowed quantification of pro-MMP-9 and MMP-9 (**d**) and pro-MMP-2 and MMP-2 (**e**) activities. Proteolytic activity was expressed as percentage of the proteolytic activity of unstimulated macrophages. Data correspond to mean values of independent experiments performed with macrophages derived from at least 5 different blood donors. Flags indicate standard error mean; *, significantly different from AGS at *p* < 0.05; **, significantly different at *p* < 0.05

Proteolysis is a critical event in the progression of cancer [33–35] and we previously reported that macrophages are the major contributors to the enhanced proteolysis found in co-cultures with cancer cells. Moreover we showed that, besides EGFR, matrix metalloproteases (MMP) were crucial for macrophage-mediated cancer cell invasion, since the presence of a broad MMP inhibitor (Galardin) was able to abolish this effect [14]. Thus, invasion assays with AGS, in the presence or absence of LPS- (CM(LPSmac)) or IL-10-stimulated (CM(IL-10mac)) macrophages, were carried out in the presence of Galardin (Fig. 6a). In fact, by inhibiting MMPs, both LPS- and IL-10-stimulated macrophage-mediated stimuli of invasion were significantly reduced.

Since MMP activity is important in terms of angiogenesis [36] and that CM from IL-10-stimulated macrophages increased the angiogenic response in the chick embryo CAM assay, we inoculated AGS cells with CM from IL-10-stimulated macrophages with Galardin (AGS + CM(IL-10-mac) + Galardin), using a control condition, in the same egg, without the inhibitor (AGS + CM(IL-10-mac)) (Fig. 6b). The presence of Galardin resulted in a slight, although statistically significant, decrease in the ability of IL-10-stimulated macrophages to induce cancer cell-mediated angiogenesis. These results indicate that MMPs are required to support cancer cell invasion provided by both macrophage populations, and to the stimulation of angiogenesis induced by CM derived from IL-10-stimulated macrophages.

### IL-10-stimulated macrophages display enhanced MMP-2 and MMP-9 activities, particularly in the presence of cancer cells

Considering the ability of a broad-MMP inhibitor to decrease cancer cell invasion and cancer cell-induced angiogenesis, we hypothesized that the distinct ability of LPS- and IL-10-stimulated macrophages in inducing these cancer cell activities could be associated to proteolytic differences. Therefore, gelatin zymography studies were conducted using CM from both macrophage-populations, when cultured alone or with cancer cells (Fig. 6c). Our results show that IL-10-stimulated macrophages (CMMat(IL-10mac)) present significantly higher pro- and active-MMP-9 activities than LPS-stimulated macrophages (CMMat(LPSmac)) (Fig. 6d). Concerning AGS cells (CMMat(AGS)), levels of MMP-9 are similar to those of LPS-stimulated but significantly lower from those of IL-10-stimulated macrophages. Regarding the proteolytic activity of invasion assays supernatants, pro- and active-MMP-9 levels in co-cultures of AGS cells with LPS-stimulated macrophages (CMMat(AGS + LPSmac) are comparable to individual population levels. Nevertheless, pro- and active-MMP-9 proteolytic activity is significantly higher in co-cultures of AGS with IL-10(CMMat(AGS+IL-10mac)) than with LPS-stimulated macrophages (CMMat(AGS + LPSmac)) (Fig. 6d). Regarding MMP-2, both pro- and active forms were significantly higher in IL-10- than in LPS-stimulated macrophages (Fig. 6e). Considering co-cultures, pro- and active-MMP-2 activity was considerably higher in AGS cells with IL-10-stimulated (CMMat(AGS + IL-10mac)) than in the corresponding condition with LPS-stimulated macrophages (CMMat(AGS + LPSmac)). In fact, no differences on MMP-2 activity were observed between LPS-stimulated macrophages alone and in co-cultures with AGS cells (Fig. 6e), suggesting that this metalloprotease is mainly produced by macrophages.

Taken together, these results indicate that differences in the stimuli of invasion provided by LPS- and IL-10-stimulated macrophages are probably related with distinct proteolytic activity profiles. Besides similar abilities in stimulating EGFR phosphorylation, IL-10-stimulated macrophages provide, in fact, higher MMP-2 and MMP-9 activities than their LPS-stimulated counterparts.

## Discussion

In this study, we investigated the role of M1 *versus* M2-like macrophages on gastric and colorectal cancer cell functions. Our results demonstrate that: 1) macrophages distinctly modulate cancer cell invasion, motility, proteolysis and angiogenesis, being M2-like macrophages (IL-10-stimulated) more efficient than their M1-like counterparts (LPS-stimulated); 2) EGFR phosphorylation is essential for both macrophage populations-mediated invasion; 3) despite differences in their pro-invasive ability, LPS- and IL-10-stimulated macrophages similarly stimulate cancer cell EGFR, c-Src, ERK1/2, Akt and p38 phosphorylation; 4) most importantly, MMPs are crucial for macrophage-mediated invasion and angiogenesis and discrepancies in the strength of these stimuli seems related with differences in the MMP activity profile of each macrophage population. As such, the higher proteolytic activity of IL-10-stimulated macrophages parallels with their higher ability to stimulate cancer cell invasion and angiogenesis.

Due to their pro-inflammatory phenotype, M1 macrophages are often associated with tumour-suppressor and cytotoxic activities. In contrast, M2-like macrophages, which display an anti-inflammatory phenotype, are generally engaged in tumour-promoting activities, in particular angiogenesis and extracellular matrix remodelling [19]. These macrophage populations also exhibit distinct molecular profiles, which are, to a certain extent, associated with the microenvironment to where monocytes are recruited and differentiated [4]. In our system, these two distinct macrophage phenotypes were obtained upon exogenous stimulation of primary human monocytes, and exhibited previously described characteristics of extremes in an activation spectrum [22]. M2-like macrophages, which share characteristics of tumour-associated macrophages (TAMs) [19], were indeed more efficient in stimulating gastric and colorectal cancer cell invasion than their LPS-stimulated counterparts. This finding is in agreement with the ability of M2-like macrophages to stimulate cancer cell migration, invasion and metastasis in other types of tumours [1]. Interestingly, LPS-stimulated macrophages also increased cancer cell invasion, although to a less extent. Accordingly, Hagemann and collaborators have previously described that LPS-stimulated macrophages were less efficient in promoting breast cancer cell invasion than naïve macrophages [37].

Cancer cell invasion results from the balance between migration and proteolysis, which facilitates the movement of cells through extracellular matrix (ECM) degraded components, allowing the release of pro-invasive and pro-motility factors entrapped within the matrix [33, 35, 38]. We have previously demonstrated that cancer cell motility is another crucial invasion-related activity stimulated by naïve macrophages [14]. In the present study, LPS- and IL-10-stimulated macrophages induced cancer cell cytoskeleton reorganization and the formation of motility-associated structures, such as lamellipodia and filopodia. Nevertheless, soluble molecules produced by IL-10-stimulated macrophages were significantly more efficient in inducing cancer cell migration than those released by LPS-stimulated macrophages. A paracrine loop involving the production of EGF by macrophages and CSF-1 by cancer cells has been demonstrated [13]. Disruption of this cellular crosstalk by blockade of EGF receptor or CSF-1 receptor signalling inhibited cancer cell migration and invasion [13]. We have also recently described the relevance of EGF-like ligands, produced by naïve macrophages, for the activation of gastric and colorectal cancer cell. EGFR and its downstream partners such as Akt, ERK1/2 and c-Src were proved to be crucial for stimulation of invasion and motility [14]. Interestingly, in the present work we demonstrate that these signalling pathways are being similarly induced by LPS- and IL-10-stimulated macrophages, despite their distinct effects on cancer cell invasion and motility. In fact, soluble factors released by both macrophage populations induced similarly EGFR, c-Src, ERK1/2, Akt, and p38 phosphorylation. Thus, our results indicate that, although critical, this pathway is not responsible for the differences in invasion-related cellular activities promoted by LPS- and IL-10-stimulated macrophages, pointing to a distinct mechanism.

MMPs are essential for tumour cell invasion through the basement membrane [35, 38] and increased expression has been positively correlated with tumour progression [36, 39]. Host cells, especially macrophages, are considered the main source of these enzymes at the tumour microenvironment [40] and we and others have recently reported that macrophage-mediated cancer cell invasion and migration are sensitive to MMP inhibition [14, 37]. In the present study, the higher MMP activity exerted by IL-10-stimulated macrophages seems to be associated with the enhancement of gastric cancer cell invasion. As previously, proteolytic activity inhibition impaired macrophage-mediated invasion [14, 37]. Taken together, our results suggest that M2-like macrophages are more efficient than their M1 counterparts in stimulating gastric cancer cell invasion, likely due to their increased MMP-2 and MMP-9 activity and higher ability to promote cancer cell migration. With increased capacity to degrade ECM components and enhanced motility and migration, the invasive ability of gastric cancer cells might be then supported.

Tumour-induced angiogenesis is essential for tumour progression since it is important for local tumour growth, survival, escape from the primary site and establishment of metastasis [41]. Macrophages are described as producers of pro-angiogenic cytokines and growth factors [42] and have already been associated with tumours neo-vascularization [43–45]. Nevertheless, different angiogenic potentials have been associated with distinct macrophage populations, with M2-like macrophages presenting pro-angiogenic features [46, 47]. Consistent with this, we observed stimulation of gastric cancer cell-induced angiogenesis *in vivo* by soluble factors secreted by M2-like macrophages. Since MMPs are crucial regulators at distinct steps of the angiogenic process [36, 48], we propose that the angiogenic stimulatory effect of M2 macrophages could be related to their enhanced MMP-2 and MMP-9 activities. However, we cannot exclude that other pro-angiogenic factors, secreted by this macrophage population might also be involved. M1 macrophages, on the other hand, were previously reported to inhibit angiogenesis in mice, even in the presence of increased levels of vascular endothelial growth factor (VEGF) [47].

## Conclusions

Overall, our results provide valuable insights into the interplay between cancer cells and two distinctly polarized macrophage populations, frequently found at the tumour microenvironment. By examining the molecular mechanisms and highlighting the importance of MMPs in such cellular crosstalk, this work offers new considerations to the establishment of more efficient therapeutic strategies, aiming to impair cancer invasion and progression.
